# Association of abdominal fat with metabolic syndrome components in overweight women: effect of menopausal status

**DOI:** 10.1186/s40101-020-00222-0

**Published:** 2020-04-19

**Authors:** Shigeharu Numao, Yasutomi Katayama, Yoshio Nakata, Tomoaki Matsuo, Masaki Nakagaichi, Kiyoji Tanaka

**Affiliations:** 1 Department of Sports and Life Sciences, National Institute of Fitness and Sports in Kanoya, 1 Shiromizu, Kanoya, Kagoshima, 891-2393 Japan; 2 Faculty of Education, Kogakukan University, 1704 Kodakushimoto, Ise, Mie 516-0016 Japan; 3 Faculty of Health and Sport Sciences, University of Tsukuba, 1-1-1 Tennodai, Tsukuba, Ibaraki, 305-8577 Japan; 4 Occupational Epidemiology Research Group, National Institute of Occupational Safety and Health, 6-21-1 Nagao, Tamaku, Kawasaki, Kanagawa 214-8585 Japan

**Keywords:** Abdominal superficial subcutaneous fat, Abdominal deep subcutaneous fat, Menopause

## Abstract

**Background:**

The association between abdominal fat distribution and metabolic syndrome (MetSyn) components by menopausal status has yet to be explicated. The purpose of this study was to examine a cross-sectional association between abdominal fat compartments and MetSyn components in pre- and post-menopausal overweight Japanese women.

**Methods:**

Of 212 overweight Japanese women, 76 pre-menopausal overweight (BMI ≥ 25) women (PreM age, 42.1 ± 5.9 years) and 87 post-menopausal overweight women (PostM: age, 56.2 ± 4.5 years) were analyzed in this study. Measurements were taken for body mass index (BMI), abdominal compartments [visceral fat (VF), subcutaneous fat (SF), superficial subcutaneous fat (SSF), and deep subcutaneous fat (DSF)], serum high-density lipoprotein cholesterol (HDLC), low-density lipoprotein cholesterol, triglycerides (TG), and fasting plasma glucose (FPG). Abdominal compartments were assessed using computed tomography.

**Results:**

No significant differences were found for BMI, SF, SSF, or DSF between the PreM and PostM. Despite this, the PreM had a significantly smaller VF area than that of the PostM. However, the difference in VF area disappeared when age was adjusted for. VFA significantly correlated with HDLC, TG, and FPG independently of menopause status.

**Conclusions:**

These results suggest that the effect of menopause status on the association between VF and MetSyn components is negligible. Abdominal subcutaneous fat compartments were not associated with MetSyn components in overweight women regardless of menopausal status.

## Background

Abdominal obesity is considered a precursor to metabolic syndrome (MetSyn) which is a cluster of risk factors leading to type 2 diabetes or cardiovascular problems [1]. Visceral fat (VF) is adipose tissue stored in the abdomen which is associated with development of MetSyn components. Changes in VF over time, such as increases and decreases during weight loss, are related to insulin resistance, hypertension, and dyslipidemia [2–7]. Abdominal subcutaneous fat (SF) also contributes to the incidence of MetSyn components [8, 9]. Interestingly, SF is separated into superficial and deep subcutaneous fat (SSF and DSF, respectively) by superficial fascia, a thin layer of connective tissue [10], and these compartments may be associated with metabolic abnormalities. Other researchers have found that SSF correlates with hepatic insulin resistance [11], insulin concentration [12, 13], and cholesterol concentration [12]. DSF is associated with glucose disposal [11, 12, 14], insulin concentration [13], cholesterol concentration [12], mean artery pressure [14], and triglyceride concentration [14]. Thus, SSF and DSF are important factors in MetSyn.

Menopause contributed to increases in body weight and changes in abdominal fat distribution [15–19]. These changes are associated with circulating sex hormone (estrogen) levels, as estrogen therapy influences abdominal fat distribution in post-menopausal women [20]. Although menopause increases VF [15–19], evidences regarding whether it affects changes to the abdominal SF distribution including SSF and DSF have been limited [19]. Sex hormone levels of overweight women differ from that of normal-weight women [21–23]. The difference in sex hormone levels could lead to altered abdominal fat distribution and the MetSyn components. Difference in sex hormone levels may change the associations between abdominal fat distribution and the MetSyn components between normal-weight and overweight women during menopause. However, because few studies have compared the association between abdominal fat distribution and the MetSyn components in pre- and post-menopausal overweight women, this association is yet to be explicated.

The purpose of the present study was to compare the abdominal fat compartments, including VF, SF, SSF, and DSF, with the MetSyn components between pre- and post-menopausal overweight women and examine the association between abdominal fat compartments and metabolic syndrome components in pre- and post-menopausal overweight women.

## Methods

### Participants

Participants were recruited from three urban communities in Japan for this study. They were recruited via advertisements in local newspapers from 1999 to 2006. There were 212 Japanese overweight women initially enrolled in this study. The participants consisted of 100 pre-menopausal overweight women and 112 post-menopausal overweight women. Overweight was defined as body mass index (BMI) ≥ 25, according to WHO classification [24]. Pre-menopause was defined as regularly menstrual cycle length, post-menopause was defined as no menses for a minimum of 1 year. Menopausal status was confirmed by a self-administered questionnaire. All participants were not involved in regular exercise training for 6 months prior to the onset of this study. Regular exercise training was defined as spontaneous physical activity, such as aerobic exercise, strength exercise, or flexible exercise, ≥ 3 session/week. The participants with a smoking habit, defined as having a smoking experiences within the past 3 years (*n* = 11), were excluded, because women smokers tend to have a 1.3 times greater VF than women non-smokers [25]. The participants with high blood glucose concentration (> 120 mg/dl) (*n* = 12), receiving hormone replacement therapy (*n* = 12) or medications affecting blood pressure, lipids, or glucose metabolism (*n* = 14) were also excluded. Finally, 76 pre-menopausal overweight women (PreM: age, 42.1 ± 5.9 years) and 87 post-menopausal overweight women (PostM: age, 56.2 ± 4.5 years) were analyzed in this study. All of them completed all measurements. Participants’ physical characteristics are shown in Table 1. Each participant heard the purpose, design, and risks associated with this study and provided written informed consent. The present study conformed to the principles outlined in the Declaration of Helsinki and was approved by the Comprehensive Human Sciences review board at the University of Tsukuba.

**Table 1:** Participants’ characteristics and metabolic syndrome components in pre and postmenopausal overweight women

	PreM (*n* = 76)	PostM (*n* = 87)	*p* value
Age (years)	42.1 ± 5.9	65.2 ± 4.5	< 0.001
Height (cm)	157.4 ± 5.3	154.3 ± 5.4	< 0.001
Weight (kg)	68.2 ± 6.4	65.2 ± 7.1	0.006
%fat (%)	39.5 ± 7.2	39.3 ± 7.2	0.861
BMI (kg/m^2^)^a^	1.44 ± 0.04	1.44 ± 0.04	0.844
WC (cm)	93.9 ± 7.5	94.3 ± 8.4	0.776
SBP (mmHg)	127 ± 15	132 ± 17	0.072
DBP (mmHg)	80 ± 11	83 ± 9	0.045
MAP (mmHg)	96.4 ± 11.5	99.3 ± 11.7	0.119
TC (mg/dl)	210.8 ± 35.9	230.7 ± 28.8	< 0.001
HDLC (mg/dl)	62.6 ± 12.9	60.9 ± 13.1	0.407
LDLC (mg/dl)	131.2 ± 32.5	148.0 ± 28.2	0.001
TG (mg/dl)^a^	1.91 ± 0.19	2.00 ± 0.19	0.006
FPG (mg/dl)	91.3 ± 7.8	93.6 ± 7.1	0.047

Values indicate mean ± SD

*PreM* pre-menopausal overweight women, *PostM* post-menopausal overweight women, *BMI* body mass index; *WC* waist circumference, *SBP* systolic blood pressure, *DBP* diastolic blood pressure, *MAP* mean artery pressure, *TC* total cholesterol, *HDLC* high-density lipoprotein cholesterol, *LDLC* low-density lipoprotein cholesterol, *FPG* fasting plasma glucose, *TG* triglycerides

^a^The values were transformed log

### Anthropometric variables

Height and weight were measured with a wall-mounted stadiometer and a digital scale, respectively. BMI was calculated as weight (kg)/height squared (m^2^). Waist circumference (WC) was measured at the umbilicus with participants standing using a non-elastic plastic measuring tape.

### Body composition measurements

Skinfold thickness was measured for the estimation of body density with an Eiken skinfold caliper at two sites, the triceps and subscapular. All skinfold thickness measurements were taken on the right side of the body, three times at each site, to the nearest 0.5 mm with the mean value recorded by a fully-trained staff member. Body density was determined with the two skinfold thickness measurements using the following predicted equation for Japanese [26].

$$ \mathrm{Body}\ \mathrm{density}=1.0897-0.00133\ \left[\mathrm{triceps}\ \mathrm{thickness}\ \left(\mathrm{mm}\right)+\mathrm{subscapular}\ \mathrm{thickness}\ \left(\mathrm{mm}\right)\right]. $$

The percentage of the amount of body fat to weight (% fat) was estimated from the body density using the Brozek et al. equation [27].

### Abdominal fat area measurements

Cross-sectional images of the abdominal fat (VF, SF, SSF, and DSF) were scanned by computed tomography (SOMATOM AR.C, Siemens, Germany) while participants were in the supine position. The manner of scanning was a single 5-mm scan with a scanning time of 5 s that was centered at the level of the umbilicus (fourth and fifth lumbar vertebrae). VF area (VFA) and the SF area (SFA) were calculated using the Fat Scan software program (N2system, Osaka, Japan) [28]. Furthermore, the boundary between the abdominal superficial subcutaneous area (SSFA) and deep subcutaneous fat area (DSFA) was defined by superficial fascia that existed in the abdominal subcutaneous fat [10]. To calculate SSFA and DSFA, fat areas that consisted of DSFA and VFA were determined by tracing, along with the superficial fascia. After that, SSFA was determined by subtracting the area of DSFA and VFA from the predetermined total abdominal fat area; and DSFA by subtracting the predetermined VFA from the area of DSFA and VFA. The intraclass correlations of these measurements in our laboratory were 0.95–0.99.

### Metabolic syndrome components

Systolic blood pressure (SBP) and diastolic blood pressure (DBP) were measured with a mercury manometer after the participants had rested at least 20 min in a sitting position. Mean artery pressure (MAP) was determined from the pressure readings using the following equation:

$$ \mathrm{MAP}=\mathrm{DBP}+\left(\mathrm{SBP}-\mathrm{DBP}\right)/3 $$

Blood samples were collected following 12-h of fasting overnight for the determination of serum total cholesterol (TC), high-density lipoprotein cholesterol (HDLC), low-density lipoprotein cholesterol (LDLC), triglycerides (TG) concentration, and fasting plasma glucose concentration (FPG).

### Blood analysis

Samples were divided into 9-ml tubes containing thrombin as the heparin neutralizing agent, and 2-ml tubes containing EDTA-2Na, heparin-Na, and sodium fluoride. The 9-ml tubes were centrifuged at 3000 g for 10 min at room temperature after 30 min of collection. The 2-ml tubes were immediately centrifuged at approximately 3000 *g* for 10 min at 4 °C. The 9-ml tube samples were used to analyze TC, HDLC, LDLC, and TG. The 2-ml tube samples were used to analyze FPG. TC was measured by cholesterol oxidase using the ·HDAOS method. HDLC and LDLC were determined by the heparin-manganese precipitation method. TG was determined by the GPO·HDAOS method, without Glycerol Blank. FPG was assayed by a glucose oxidase method.

### Statistical analysis

All data were expressed as the mean ± standard deviation (SD). Based on a previous study [15], sample size was calculated to detect a moderate to large effect (Cohen’s *d* = 0.49–0.92). It was determined that an estimated sample size of 40–134 would be required to have approximately an 80% power needed to detect a moderate to large effect at 0.05 significance. The assumption of normal distribution was confirmed using the Kolmogorov–Smirnov test and skewness. The variables (BMI and TG) were not normally distributed and were log-transformed. *F* test was used to confirm the assumption of homoscedasticity. To compare the characteristics and MetSyn components between the PreM and PostM, an unpaired *t* test was used. Analysis of the covariance (ANCOVA) was used to test differences in the abdominal fat distribution adjusted for age, and the MetSyn components between the PreM and PostM adjusted for age or VFA. Pearson product-moment and partial correlation coefficients were calculated to determine the association between different abdominal fat areas and the MetSyn components. A false discovery rate (FDR) adjustment was used to adjust the significance level of Pearson product-moment and partial correlation coefficients for multiple comparisons [29]. In the sensitivity analyses, outliers were identified and excluded based on the cutoff for leverage values [30]. Multiple regression analysis was performed with MetSyn components as dependent variable and menopause status (pre-menopause 0 and post-menopause 1) and abdominal fat compartments as independent variables in all participants. Sensitivity analyses of multiple regression analysis did not performed, because outliers over the cutoff for leverage values [30] were not detected. The effect size (ES) was judged with Cohen’s *d* (small ≥ 0.20, medium ≥ 0.50, or large ≥ 0.80) for comparison of the characteristics and MetSyn components between PreM and PostM. Statistical significance was set at the 0.05 level of confidence.

## Results

### Physical characteristics

Participants’ physical characteristics are summarized in Table 1. No significant differences were observed in BMI, % fat, and WC. There were no differences found for SBP, and MAP between the PreM and PostM. Whereas TC, LDLC, and TG were significantly higher in the PostM than in the PreM (95% CI 9.9–29.9, ES 0.62; 95% CI 7.4–26.1, ES 0.56; and 95% CI 0.03–0.15, ES 0.48, respectively). Following adjustment for age, significant differences in TC, LDLC, and TG disappeared (95% CI − 16.3–16.7, ES 0.00; 95% CI − 13.9–17.3, ES 0.04; and 95% CI − 0.06–0.14, ES 0.16, respectively). Moreover, once adjusted for VFA, TC, and LDLC remained significantly different (95% CI 6.8–27.6, ES 0.53 and 95% CI 3.1–22.1, ES 0.47, respectively), whereas the difference in TG became insignificant (95% CI − 0.01–0.11, ES 0.33).

### Abdominal fat areas

Abdominal fat areas are summarized in Table 2. VFA was significantly lower in the PreM rather than the PostM (95% CI 10.4–34.5, ES 0.58). However, following adjustment for age, significant differences in VFA disappeared (95% CI − 17.0–22.5, ES 0.05). No significant differences were found for SFA, SSFA, and DSFA (95% CI − 19.7–17.2, ES 0.02, 95% CI − 16.2–2.2, ES 0.24, and 95% CI − 6.7–18.3, ES 0.15, respectively) between the PreM and PostM.

**Table 2:** Abdominal fat area in pre and postmenopausal overweight women

	PreM (*n* = 76)	PostM (*n* = 87)	*p* value	Adjusted *p* value^a^
TFA (cm^2^)	355.9 ± 75.8	377.1 ± 74.5	0.075	0.735
SFA (cm^2^)	266.0 ± 61.5	264.8 ± 57.8	0.894	0.546
SSFA (cm^2^)	138.3 ± 30.3	131.3 ± 29.1	0.133	0.759
DSFA (cm^2^)	127.7 ± 41.7	133.5 ± 38.9	0.361	0.264
VFA (cm^2^)	89.9 ± 34.7	112.3 ± 42.1	< 0.001	0.782

Values indicate mean ± SD

*PreM* pre-menopausal overweight women, *PostM* post-menopausal overweight women, *TFA* total abdominal fat area, *SFA* subcutaneous fat area, *SSFA* superficial subcutaneous fat area, *DSFA* deep subcutaneous fat area, *VFA* visceral fat area

^a^Adjusted for age

### Correlation between the abdominal fat area and the metabolic syndrome components

Correlation coefficients and partial correlation coefficients between the abdominal fat areas and the MetSyn components were summarized in Tables 3; Table S1 and Table S2. VFA exhibited significant correlations with TC (*r* = 0.21, 95%CI 0.06–0.35), HDLC (*r* = − 0.19, 95%CI − 0.33 to − 0.04), LDLC (*r* = 0.23, 95%CI 0.08–0.37), and TG (*r* = 0.36, 95%CI 0.22–0.49) in all participants. In the PreM, VFA was significantly correlated with TC (*r* = 0.28, 95%CI 0.06–0.48), LDLC (*r* = 0.38, 95%CI 0.17–0.56), and TG (*r* = 0.37, 95%CI 0.16–0.55). Following adjustment for age and BMI, VFA exhibited significant correlations with HDLC (*r* = − 0.31, 95%CI − 0.50 to − 0.09), LDLC (*r* = 0.28, 95%CI 0.06–0.48), and TG (*r* = 0.35, 95%CI 0.14–0.53). In PostM, VFA was significantly correlated with TG (*r* = 0.27, 95%CI 0.06–0.45). Following adjustment for age and BMI, the significance was maintained (Table S1, and Table S2). However, TFA, SFA, SSFA, and DSFA did not significantly correlate with any of the MetSyn components in both the PreM and PostM even after adjustment for age and BMI. In the sensitivity analyses, the significance of correlations did not change in either the PreM or PostM (Supporting information Tables S3, S4, and S5).

**Table 3:** Pearson product-moment correlation coefficients between abdominal fat areas and metabolic syndrome components in overweight women

		SBP	DBP	MAP	TC	HDLC	LDLC	TG	FPG
TFA	All	0.17	0.17	0.14	0.08	− 0.05	0.09	0.15	0.18
	PreM	0.17	0.28	0.18	0.17	− 0.02	0.22	0.13	0.20
	PostM	0.14	0.01	0.09	− 0.09	− 0.05	− 0.11	0.12	0.13
SFA	All	0.15	0.10	0.12	− 0.39	0.06	− 0.04	− 0.05	0.11
	PreM	0.19	0.24	0.18	0.05	0.12	0.05	− 0.05	0.19
	PostM	0.12	− 0.04	0.09	− 0.14	0.01	− 0.14	− 0.04	0.04
SSFA	All	0.06	0.05	0.11	− 0.04	0.06	− 0.01	− 0.08	0.09
	PreM	0.19	0.16	0.21	− 0.02	0.10	0.02	− 0.08	0.18
	PostM	− 0.01	− 0.02	0.02	− 0.01	− 0.02	0.04	− 0.04	0.05
DSFA	All	0.17	0.11	0.09	− 0.02	0.06	− 0.06	− 0.01	0.10
	PreM	0.19	0.23	0.12	0.09	0.11	0.06	− 0.02	0.15
	PostM	0.19	− 0.05	0.08	− 0.20	0.03	− 0.24	− 0.03	0.02
VFA	All	0.10	0.17	0.10	0.21*	− 0.19 *	0.23*	0.36*	0.18
	PreM	0.03	0.19	0.08	0.28 *	− 0.27	0.38*	0.37*	0.11
	PostM	0.08	0.09	0.07	0.03	− 0.11	− 0.01	0.27*	0.17

*SBP* systolic blood pressure, *DBP* diastolic blood pressure, *MAP* mean artery pressure, *TC* total cholesterol, *HDLC* high-density lipoprotein cholesterol, *LDLC* low-density lipoprotein cholesterol, *FPG* fasting plasma glucose, *TG* triglycerides, *TFA* total abdominal fat area, *SFA* subcutaneous fat area, *SSFA* superficial subcutaneous fat area, *DSFA* deep subcutaneous fat area, *VFA* visceral fat area. *PreM* pre-menopausal overweight women, *PostM* post-menopausal overweight women

*There was a significant correlation after a false discovery rate adjustment

In multiple regression analysis (Table 4), menopause status was not independent predictor of any of the MetSyn components. VFA was the independent predictor of HDLC (β = − 0.19, 95%CI − 0.11 to − 0.01), TG (β = 0.36, 95%CI 0.001–0.002), and FPG (β = 0.18, 95%CI 0.005–0.06). Other abdominal fat compartments were not significantly correlated with the MetSyn components.

**Table 4:** Multiple regression analysis assessing the association between abdominal fat compartments and metabolic syndrome components

Dependent variable	Model	β	β_SE_	Standard β	*p* value	*R*^2^
SBP	Model 1: age	0.45	0.14	0.24	0.002	0.06
	Model 2: age	0.44	0.14	0.24	0.002	0.09
	BMI	86.5	35.9	0.18	0.017	
DBP	Model 1: age	0.26	0.09	0.23	0.004	0.05
	Model 2: age	0.25	0.09	0.23	0.004	0.08
	BMI	45.4	21.8	0.16	0.039	
MAP	Model 1: BMI	68.3	25.3	0.21	0.008	0.04
	Model 2: BMI	67.6	25.0	0.21	0.008	0.08
	age	0.24	0.10	0.18	0.017	
TC	Model 1: age	1.41	0.28	0.37	< 0.001	0.13
HDLC	Model 1: VFA	− 0.06	0.03	− 0.19	0.018	0.03
LDLC	Model 1: age	1.15	0.27	0.32	< 0.001	0.10
TG	Model 1: VFA	0.002	0.001	0.36	< 0.001	0.13
FBG	Model 1: VFA	0.03	0.01	0.18	0.022	0.03

*SBP* systolic blood pressure, *DBP* diastolic blood pressure, *MAP* mean artery pressure, *TC* total cholesterol, *HDLC* high-density lipoprotein cholesterol, *LDLC* low-density lipoprotein cholesterol, *FPG* fasting plasma glucose, *TG* triglycerides, *BMI* body mass index, *VFA* visceral fat area

## Discussion

The purpose of the present study was to compare the abdominal fat compartments, including VF, SF, SSF, and DSF, with the MetSyn components between pre- and post-menopausal overweight women and to determine the differences associated with the abdominal fat compartments and the MetSyn components between pre- and post-menopausal overweight women. Our results indicated that although VFA was higher in the PostM than in the PreM, the difference in VFA disappeared after adjustment for age. SFA, SSFA, and DSFA were similar between the PreM and PostM. Furthermore, TC, LDLC, and TG were significantly higher in the PostM than in the PreM, while no significant difference in TC and LDLC were found after adjustment for age. Multiple regression revealed menopause was not an independent predictor of MetSyn components, while VFA significantly associated with HDLC, FBG, and TG regardless of menopause status. These results suggest that age rather than menopause status affects the association between abdominal fat compartment and MetSyn components.

Menopause is believed to influence abdominal fat distribution as VF increases rapidly (2.6 times) following menopause [31]. VF was higher in post-menopausal women than pre-menopausal women, even when the BMI measurements were similar [16–18]. Our findings expand upon the results of these studies. However, following adjustment for age, significant differences between PreM and PostM in VFA disappeared in our study. This suggests that the difference in VF between pre-menopausal and post-menopausal overweight women is not necessary explained by menopause alone. Aging is also attributed to the difference in VF between pre-menopausal and post-menopausal overweight women as shown by meta-analysis [32].

To our knowledge, there has been one study that has compared the abdominal subcutaneous fat compartments (SSF and DSF) between pre- and post-menopausal women [19]. Lovejoy et al. [19] reported that SSF and DSF area did not differ between pre- and post-menopausal women at baseline and at follow-up (after 4 years). Our results also indicated that SFA, SSFA, and DSFA were not significantly different between the pre- and postmenopausal overweight women. However, the selection of participants to target overweight women may influence the results. Especially, a similar BMI in pre- and post-menopausal overweight women may contribute to abdominal subcutaneous fat compartments. In previous study [19], BMI was similar between pre-menopausal and post-menopausal women. Because BMI is generally higher in post-menopausal women than in pre-menopausal women [32], our results cannot apply to the populations other than pre-menopausal and postmenopausal overweight women with a similar BMI.

MetSyn components often deteriorated following menopause [2, 16, 17]. One of the reasons for this deterioration appears to be the accumulation of excess VF that occurred after menopause [16, 17]. In our study, post-menopausal overweight women were significantly higher in VFA together with TC, LDLC, and TG levels than the pre-menopausal overweight women. Following adjustments for VFA, significant differences between the pre- and post-menopausal overweight women were found in the TC and LDLC, and the difference in TG disappeared. This suggests that the accumulation of VF contributed to the deterioration of the TG levels. In contrast, the deterioration of the TC and LDLC in post-menopausal overweight women could be influenced by other factors than VF, such as the stages of menopause or aging-related disorders that complicated the TC and LDLC levels. For instance, in our study, significant differences were found in TC and LDLC between the PreM and PostM until these variables were adjusted for age, and subsequently these differences disappeared. Similar results were found in Framingham study that reported increases in TC and LDLC had occurred with aging [33]. A current meta-analysis showed that TC and LDLC were significantly higher in post-menopausal women than in pre-menopausal women [34]. The effects of aging and menopause on the difference in lipoproteins between pre-menopausal and post-menopausal women were also reported [34]. Thus, it seems that metabolic changes mediated by age could be the cause of deterioration in TC and LDLC levels.

It has been widely known that abdominal fat, especially VF, is related to MetSyn components. However, the association between abdominal fat compartments and the MetSyn components might instead be altered by menopause. Zamboni et al. [16] showed that postmenopausal obese women exhibited fewer significant correlations between abdominal fat (abdominal fat area, visceral fat area, subcutaneous area, and the ratio of the visceral and subcutaneous fat area) and the MetSyn components than the pre-menopausal obese women. Our study found that in the separate correlation analysis, pre-menopausal women had significant correlations between VFA and TC, LDLC, and TG, while post-menopausal women had an only significant correlation between VFA and TG. However, in the multiple regression analysis using all participants, menopause status was not adopted as an independent predictor. This suggests menopause status do not necessarily affect the association between abdominal fat and the MetSyn components. Nevertheless, VFA was the independent predictor of HDLC, TG, and FBG. This indicates VF plays an important role in MetSyn components in overweight women regardless of menopause status.

Several previous studies have reported that SSF and DSF were associated with the MetSyn components [11–14]. However, our results are not in agreement with these results. This discrepancy may be explained by the gender of the participants. SSF and DSF were correlated positively with hepatic insulin resistance in men with type 2 diabetes, while this correlation was not observed in women with type 2 diabetes [11]. The relationship between SSF, DSF, and the MetSyn components for both men and women has been observed, but the effect of SSF and DSF on the MetSyn components is much weaker in women than in men [13]. Moreover, SSF and DSF were not associated with glucose disposal in premenopausal women [35] or lipid-lipoprotein in women [36]. These results suggest that different metabolic characteristics exist for SSF and DSF between men and women. In fact, the differences noted in the lipolytic response between SSF and DSF in men has been evaluated with both in vivo [37] and in vitro studies [38]. Although these results are not yet available for women, gender differences involved with the metabolic characteristics for SSF and DSF might be causing the differences found in the levels of SSF, DSF, and the MetSyn components between men and women.

There are several limitations of this study. First, our participants were Japanese women. Japanese adults have a propensity for greater VF than SF compared with other ethnic populations [39, 40]. Therefore, our data likely does not apply to other ethnic populations. Second, menopause status could not be confirmed by several hormones [41]. However, none of pre-menopausal overweight women had irregular menstrual cycle length, and none of post-menopausal overweight women had menses for a minimum of 1 year in the present study. Third, post-menopausal time periods in post-menopausal women are not taken into accounts. It is possible that post-menopausal time periods predict the body fat mass and trunk fat [2, 19, 42]. Forth, as our study design was a cross-sectional study, it is unclear whether the effects of longitudinal change in the abdominal subcutaneous fat compartments, including SSF and DSF on the MetSyn components in the pre- and post-menopausal overweight women. Further studies are also required to determine that changes to the abdominal subcutaneous fat compartments contributed to the MetSyn components in pre- and post-menopausal overweight women.

## Conclusions

Despite similar BMI between the pre- and post-menopausal overweight women, post-menopausal women possessed more significant accumulation of VF than pre-menopausal women. However, the difference in VF between pre-menopausal and post-menopausal overweight women is likely to be due to aging rather than menopause. On the other hand, VF was associated with the MetSyn components in the overweight women independently of menopause status. However, the contribution of abdominal subcutaneous fat compartments to MetSyn components appeared to be minimal in both pre- and post-menopausal overweight women.

## Supplementary information

**Additional file 1: Table S1.** Partial correlation coefficients between abdominal fat areas and metabolic syndrome components in pre-menopausal overweight women. **Table S2.** Partial correlation coefficients between abdominal fat areas and metabolic syndrome components in post-menopausal overweight women. **Table S3.** Pearson product-moment correlation coefficients between abdominal fat areas and metabolic syndrome components in overweight women, excluding participants with high leverage value. **Table S4.** Partial correlation coefficients between abdominal fat areas and metabolic syndrome components in pre-menopausal overweight women, excluding participants with high leverage value. **Table S5.** Partial correlation coefficients between abdominal fat areas and metabolic syndrome components in post-menopausal overweight women, excluding participants with high leverage value.

## Data Availability

All data generated or analyzed during this study are included in this published article.

## Abbreviations

MetSyn: Metabolic syndrome
VF: Visceral fat
SF: Subcutaneous fat
SSF: Superficial subcutaneous fat
DSF: Deep subcutaneous fat
BMI: Body mass index
PreM: Pre-menopausal overweight women
PostM: Post-menopausal overweight women
WC: Waist circumference
%fat: Percentage of body fat
VFA: Visceral fat area
SFA: Subcutaneous fat area
SSFA: Abdominal superficial subcutaneous area
DSFA: Abdominal deep subcutaneous fat area
SBP: Systolic blood pressure
DBP: Diastolic blood pressure
MAP: Mean artery pressure
TC: Total cholesterol
HDLC: High-density lipoprotein cholesterol
LDLC: Low-density lipoprotein cholesterol
TG: Triglycerides
FPG: Fasting plasma glucose
SD: Standard deviation
ANCOVA: Analysis of the covariance
ES: Effect size
