# Supplementary material for: Association of abdominal fat with metabolic syndrome components in overweight women: effect of menopausal status
Source: J Physiol Anthropol. 2020 Apr 19;39:12. doi: 10.1186/s40101-020-00222-0 (PMC7168819; doi:10.1186/s40101-020-00222-0)
Supplement: Supplementary file 1 — Additional file 1: Table S1. Partial correlation coefficients between abdominal fat areas and metabolic syndrome components in pre-menopausal overweight women. Table S2. Partial correlation coefficients between abdominal fat areas and metabolic syndrome components in post-menopausal overweight women. Table S3. Pearson product-moment correlation coefficients between abdominal fat areas and metabolic syndrome components in overweight women, excluding participants with high leverage value. Table S4. Partial correlation coefficients between abdominal fat areas and metabolic syndrome components in pre-menopausal overweight women, excluding participants with high leverage value. Table S5. Partial correlation coefficients between abdominal fat areas and metabolic syndrome components in post-menopausal overweight women, excluding participants with high leverage value. [file 40101_2020_222_MOESM1_ESM.docx]

**Supplementary Materials**

**Table S1**. Partial correlation coefficients between abdominal fat areas and metabolic syndrome components in pre-menopausal overweight women.

|  |  | SBP | DBP | MAP | TC | HDLC | LDLC | TG | FPG |
| --- | --- | --- | --- | --- | --- | --- | --- | --- | --- |
| TFA | Age | 0.14 (0.02) | 0.26 (0.07) | 0.17 (0.03) | 0.15 (0.02) | -0.04 (0.001) | 0.20 (0.04) | 0.11 (0.01) | 0.20 (0.04) |
|  | BMI | 0.11 (0.01) | 0.18 (0.03) | 0.12 (0.01) | 0.15 (0.02) | 0.07 (0.005) | 0.11 (0.01) | 0.12 (0.01) | 0.07 (0.005) |
|  | Age and BMI | 0.10 (0.01) | 0.17 (0.03) | 0.12 (0.01) | 0.14 (0.02) | 0.06 (0.004) | 0.11 (0.01) | 0.11 (0.01) | 0.07 (0.005) |
| SFA | Age | 0.23 (0.05) | 0.27 (0.07) * | 0.21 (0.04) | 0.07 (0.005) | 0.13 (0.02) | 0.07 (0.005) | -0.04 (0.002) | 0.20 (0.04) |
|  | BMI | 0.14 (0.02) | 0.14 (0.02) | 0.13 (0.02) | -0.01 (0.0001) | 0.24 (0.06) | -0.09 (0.008) | -0.12 (0.01) | 0.07 (0.005) |
|  | Age and BMI | 0.21 (0.04) | 0.19 (0.04) | 0.18 (0.03) | 0.04 (0.002) | 0.26 (0.07) | -0.06 (0.004) | -0.09 (0.008) | 0.08 (0.006) |
| SSFA | Age | 0.22 (0.05) | 0.18 (0.03) | 0.23 (0.05) | -0.01 (0.0001) | 0.11 (0.01) | 0.04 (0.002) | -0.07 (0.005) | 0.18 (0.03) |
|  | BMI | 0.15 (0.02) | 0.05 (0.003) | 0.16 (0.03) | -0.09 (0.008) | 0.19 (0.04) | -0.10 (0.01) | -0.15 (0.02) | 0.07 (0.005) |
|  | Age and BMI | 0.20 (0.04) | 0.09 (0.008) | 0.20 (0.04) | -0.06 (0.004) | 0.20 (0.04) | -0.08 (0.006) | -0.12 (0.01) | 0.09 (0.008) |
| DSFA | Age | 0.17 (0.03) | 0.27 (0.07) * | 0.15 (0.02) | 0.11 (0.01) | 0.12 (0.01) | 0.08 (0.006) | -0.01 (0.0001) | 0.16 (0.03) |
|  | BMI | 0.09 (0.008) | 0.15 (0.02) | 0.06 (0.004) | 0.05 (0.003) | 0.18 (0.03) | -0.05 (0.003) | -0.06 (0.004) | 0.05 (0.003) |
|  | Age and BMI | 0.14 (0.02) | 0.19 (0.04) | 0.10 (0.01) | 0.09 (0.008) | 0.21(0.04) | -0.02 (0.0004) | -0.04 (0.002) | 0.06 (0.004) |
| VFA | Age | -0.10 (0.01) | 0.10 (0.01) | -0.02 (0.0004) | 0.20 (0.04) | -0.33 (0.11) * | 0.33 (0.11) * | 0.34 (0.12) * | 0.08 (0.006) |
|  | BMI | -0.03 (0.0009) | 0.10 (0.01) | 0.02 (0.0004) | 0.27 (0.07) | -0.25 (0.06) | 0.33 (0.11) * | 0.38 (0.14) * | 0.01 (0.0001) |
|  | Age and BMI | -0.16 (0.03) | 0.01 (0.0001) | -0.08 (0.006) | 0.19 (0.04) | -0.31 (0.10) * | 0.28 (0.08) * | 0.35 (0.12) * | -0.02 (0.0004) |

Value in bracket is presented as R^2^. BMI, body mass index; SBP, systolic blood pressure; DBP, diastolic blood pressure; MAP, mean artery pressure; TC, total cholesterol; HDLC, high-density lipoprotein cholesterol; LDLC, low-density lipoprotein cholesterol; FPG, fasting plasma glucose; TG, triglycerides; TFA, total abdominal fat area; SFA, subcutaneous fat area; SSFA, superficial subcutaneous fat area; DSFA, deep subcutaneous fat area; VFA, visceral fat area. *There was a significant correlation after a false discovery rate adjustment

**Table S2**. Partial correlation coefficients between abdominal fat areas and metabolic syndrome components in post-menopausal overweight women.

|  |  | SBP | DBP | MAP | TC | HDLC | LDLC | TG | FPG |
| --- | --- | --- | --- | --- | --- | --- | --- | --- | --- |
| TFA | Age | 0.14 (0.02) | 0.01 (0.0001) | 0.09 (0.008) | -0.10 (0.01) | -0.06 (0.004) | -0.12 (0.01) | 0.12 (0.01) | 0.13 (0.02) |
|  | BMI | 0.11(0.01) | -0.06 (0.004) | -0.10 (0.01) | -0.04 (0.002) | -0.13 (0.02) | -0.06 (0.004) | 0.18 (0.03) | 0.09 (0.008) |
|  | Age and BMI | -0.01 (0.0001) | -0.06 (0.004) | -0.10 (0.01) | -0.06 (0.004) | -0.13 (0.02) | -0.07 (0.005) | 0.16 (0.03) | 0.08 (0.006) |
| SFA | Age | 0.13 (0.02) | -0.04 (0.002) | 0.07 (0.005) | -0.13 (0.02) | 0.01(0.0001) | -0.13 (0.02) | -0.04 (0.002) | 0.04 (0.002) |
|  | BMI | -0.01(0.0001) | -0.12 (0.01) | -0.11 (0.01) | -0.10 (0.01) | -0.05 (0.003) | -0.09 (0.008) | -0.01(0.0001) | -0.02 (0.0004) |
|  | Age and BMI | -0.01(0.0001) | -0.12 (0.01) | -0.11 (0.01) | -0.10 (0.01) | -0.05 (0.003) | -0.09 (0.008) | -0.01(0.0001) | -0.02 (0.0004) |
| SSFA | Age | -0.01(0.0001) | -0.02 (0.0004) | 0.02 (0.0004) | 0.01(0.0001) | -0.02 (0.0004) | 0.05 (0.003) | -0.04 (0.002) | 0.06 (0.004) |
|  | BMI | -0.14 (0.02) | -0.08 (0.006) | -0.10 (0.01) | 0.05 (0.003) | -0.08 (0.006) | 0.11 (0.01) | -0.01 (0.0001) | 0.01(0.0001) |
|  | Age and BMI | -0.14 (0.02) | -0.08 (0.006) | -0.10 (0.01) | 0.05 (0.003) | -0.08 (0.006) | 0.11 (0.01) | -0.01 (0.0001) | 0.02 (0.0004) |
| DSFA | Age | 0.19 (0.04) | -0.05 (0.003) | 0.08 (0.006) | -0.19 (0.04) | 0.03 (0.0009) | -0.23 (0.05) | -0.03 (0.0009) | 0.02 (0.0004) |
|  | BMI | 0.10 (0.01) | -0.11 (0.01) | -0.08 (0.006) | -0.17 (0.03) | -0.01 (0.0001) | -0.21 (0.04) | -0.01 (0.0001) | -0.03 (0.0009) |
|  | Age and BMI | 0.10 (0.01) | -0.11 (0.01) | -0.08 (0.006) | -0.17 (0.03) | -0.01 (0.0001) | -0.21 (0.04) | -0.01 (0.0001) | -0.03 (0.0009) |
| VFA | Age | 0.07 (0.005) | 0.09 (0.008) | 0.07 (0.005) | 0.01 (0.0001) | -0.11 (0.01) | -0.03 (0.0009) | 0.27 (0.07) * | 0.17 (0.03) |
|  | BMI | 0.02 (0.0004) | 0.06 (0.004) | -0.01 (0.0001) | 0.06 (0.004) | -0.14 (0.02) | 0.02 (0.0004) | 0.29 (0.08) * | 0.15 (0.02) |
|  | Age and BMI | 0.01 (0.0001) | 0.06 (0.004) | -0.02 (0.0004) | 0.03 (0.0009) | -0.15 (0.02) | 0.01 (0.0001) | 0.30 (0.09) * | 0.14 (0.02) |

Value in bracket is presented as R^2^. BMI, body mass index; SBP, systolic blood pressure; DBP, diastolic blood pressure; MAP, mean artery pressure; TC, total cholesterol; HDLC, high-density lipoprotein cholesterol; LDLC, low-density lipoprotein cholesterol; FPG, fasting plasma glucose; TG, triglycerides; TFA, total abdominal fat area; SFA, subcutaneous fat area; SSFA, superficial subcutaneous fat area; DSFA, deep subcutaneous fat area; VFA, visceral fat area. *There was a significant correlation after a false discovery rate adjustment

**Table S3**. Pearson product-moment correlation coefficients between abdominal fat areas and metabolic syndrome components in overweight women, excluding participants with high leverage value.

|  |  | SBP | DBP | MAP | TC | HDLC | LDLC | TG | FPG |
| --- | --- | --- | --- | --- | --- | --- | --- | --- | --- |
| TFA | All (n = 160) | 0.19 (0.04) | 0.18 (0.03) | 0.16 (0.03) | 0.07 (0.005) | -0.08 (0.006) | 0.08 (0.006) | 0.17 (0.03) | 0.18 (0.03) |
|  | PreM (n =74) | 0.19 (0.04) | 0.27 (0.07) | 0.20 (0.04) | 0.14 (0.02) | 0.04 (0.002) | 0.19 (0.04) | 0.09 (0.008) | 0.21 (0.04) |
|  | PostM (n = 85) | 0.14 (0.02) | 0.01 (0.0001) | 0.11 (0.01) | -0.13 (0.02) | -0.09 (0.008) | -0.14 (0.02) | 0.13 (0.02) | 0.14 (0.02) |
| SFA | All (n = 160) | 0.12 (0.01) | 0.11 (0.01) | 0.11 (0.01) | -0.08 (0.006) | 0.07 (0.005) | -0.08 (0.006) | -0.07 (0.005) | 0.09 (0.008) |
|  | PreM (n =74) | 0.20 (0.04) | 0.25 (0.06) | 0.20 (0.04) | 0.04 (0.002) | 0.17 (0.03) | 0.03 (0.0009) | -0.09 (0.008) | 0.16 (0.03) |
|  | PostM (n = 85) | 0.10 (0.01) | -0.05 (0.003) | 0.08 (0.006) | -0.20 (0.04) | -0.01 (0.0001) | -0.20 (0.04) | -0.04 (0.002) | 0.05 (0.003) |
| SSFA | All (n = 162) | 0.07 (0.005) | 0.06 (0.004) | 0.10 (0.01) | -0.11 (0.01) | 0.04 (0.002) | -0.04 (0.002) | -0.12 (0.01) | 0.07 (0.005) |
|  | PreM (n =74) | 0.18 (0.03) | 0.18 (0.03) | 0.18 (0.03) | -0.03 (0.0009) | 0.09 (0.008) | 0.03 (0.0009) | -0.09 (0.008) | 0.16 (0.03) |
|  | PostM (n = 84) | 0.09 (0.008) | 0.01 (0.0001) | 0.12 (0.01) | -0.16 (0.03) | -0.04 (0.002) | -0.09 (0.008) | -0.07 (0.005) | 0.08 (0.006) |
| DSFA | All (n = 158) | 0.10 (0.01) | 0.10 (0.01) | 0.04 (0.002) | -0.06 (0.004) | 0.03 (0.0009) | -0.07 (0.005) | -0.04 (0.002) | 0.04 (0.002) |
|  | PreM (n =74) | 0.18 (0.03) | 0.26 (0.07) | 0.15 (0.02) | 0.08 (0.006) | 0.14 (0.02) | 0.04 (0.002) | -0.03 (0.0009) | 0.18 (0.03) |
|  | PostM (n = 84) | 0.17 (0.03) | -0.05 (0.003) | 0.06 (0.004) | -0.30 (0.09) * | 0.02 (0.0004) | -0.33 (0.11) * | -0.04 (0.002) | 0.02 (0.0004) |
| VFA | All (n = 160) | 0.15 (0.02) | 0.18 (0.03) | 0.12 (0.01) | 0.21 (0.04) * | -0.21 (0.04) * | 0.23 (0.05) * | 0.34 (0.12) * | 0.19 (0.04) |
|  | PreM (n =74) | 0.05 (0.003) | 0.20 (0.04) | 0.09 (0.008) | 0.27 (0.07) | -0.26 (0.07) | 0.38 (0.14) * | 0.37 (0.14) * | 0.14 (0.02) |
|  | PostM (n = 83) | 0.18 (0.03) | 0.11 (0.01) | 0.17 (0.03) | 0.08 (0.006) | -0.16 (0.03) | 0.05 (0.003) | 0.28 (0.08) * | 0.24 (0.06) |

Value in bracket is presented as R^2^. PreM, premenopausal women; PostM, postmenopausal women; SBP, systolic blood pressure; DBP, diastolic blood pressure; MAP, mean artery pressure; TC, total cholesterol; HDLC, high-density lipoprotein cholesterol; LDLC, low-density lipoprotein cholesterol; FPG, fasting plasma glucose; TG, triglycerides; TFA, total abdominal fat area; SFA, subcutaneous fat area; SSFA, superficial subcutaneous fat area; DSFA, deep subcutaneous fat area; VFA, visceral fat area. *There was a significant correlation after a false discovery rate adjustment

**Table S4**. Partial correlation coefficients between abdominal fat areas and metabolic syndrome components in pre-menopausal overweight women, excluding participants with high leverage value.

|  |  | SBP | DBP | MAP | TC | HDLC | LDLC | TG | FPG |
| --- | --- | --- | --- | --- | --- | --- | --- | --- | --- |
| TFA | Age | 0.17 (0.03) | 0.27 (0.07) | 0.18 (0.03) | 0.11 (0.01) | 0.02 (0.0004) | 0.17 (0.03) | 0.08 (0.006) | 0.20 (0.04) |
| (n=74) | BMI | 0.14 (0.02) | 0.20 (0.04) | 0.14 (0.02) | 0.13 (0.02) | 0.11 (0.01) | 0.09 (0.008) | 0.09 (0.008) | 0.08 (0.006) |
|  | Age and BMI | 0.13 (0.02) | 0.19 (0.04) | 0.13 (0.02) | 0.12 (0.01) | 0.10 (0.01) | 0.09 (0.008) | 0.09 (0.008) | 0.08 (0.006) |
| SFA | Age | 0.26 (0.07) | 0.27 (0.07) | 0.24 (0.06) | 0.07 (0.005) | 0.20 (0.04) | 0.06 (0.004) | -0.08 (0.006) | 0.17 (0.03) |
| (n=74) | BMI | 0.11 (0.01) | 0.10 (0.01) | 0.09 (0.008) | -0.02 (0.0004) | 0.24 (0.06) | -0.10 (0.01) | -0.11 (0.01) | 0.10 (0.01) |
|  | Age and BMI | 0.20 (0.04) | 0.16 (0.03) | 0.15 (0.02) | 0.04 (0.002) | 0.29 (0.08) | -0.06 (0.004) | -0.09 (0.008) | 0.11 (0.01) |
| SSFA | Age | 0.24 (0.06) | 0.22 (0.05) | 0.22 (0.05) | 0.01 (0.0001) | 0.12 (0.01) | 0.05 (0.003) | -0.08 (0.006) | 0.17 (0.03) |
| (n=74) | BMI | 0.14 (0.02) | 0.08 (0.006) | 0.12 (0.01) | -0.09 (0.008) | 0.14 (0.02) | -0.09 (0.008) | -0.13 (0.02) | 0.06 (0.004) |
|  | Age and BMI | 0.21(0.04) | 0.13 (0.02) | 0.18 (0.03) | -0.05 (0.003) | 0.18 (0.03) | -0.06 (0.004) | -0.11 (0.01) | 0.07 (0.005) |
| DSFA | Age | 0.19 (0.04) | 0.27 (0.07) | 0.16 (0.03) | 0.07 (0.005) | 0.14 (0.02) | 0.05 (0.003) | -0.03 (0.0009) | 0.18 (0.03) |
| (n=74) | BMI | 0.07 (0.005) | 0.13 (0.02) | 0.03 (0.0009) | 0.03 (0.0009) | 0.18 (0.03) | -0.06 (0.004) | -0.04 (0.002) | 0.11 (0.01) |
|  | Age and BMI | 0.12 (0.01) | 0.16 (0.03) | 0.06 (0.004) | 0.07 (0.005) | 0.19 (0.04) | -0.03 (0.0009) | -0.02 (0.0004) | 0.12 (0.01) |
| VFA | Age | -0.10 (0.01) | 0.09 (0.008) | -0.02 (0.0004) | 0.17 (0.03) | -0.33 (0.11) * | 0.31 (0.10) * | 0.33 (0.11) * | 0.11 (0.01) |
| (n=74) | BMI | -0.02 (0.0004) | 0.10 (0.01) | 0.02 (0.0004) | 0.26 (0.07) | -0.25 (0.06) | 0.33 (0.11) * | 0.37 (0.14) * | 0.06 (0.004) |
|  | Age and BMI | -0.17 (0.03) | -0.01 (0.0001) | -0.09 (0.008) | 0.17 (0.03) | -0.32 (0.10) * | 0.28 (0.08) * | 0.35 (0.12) * | 0.04 (0.002) |

Value in bracket is presented as R^2^. BMI, body mass index; SBP, systolic blood pressure; DBP, diastolic blood pressure; MAP, mean artery pressure; TC, total cholesterol; HDLC, high-density lipoprotein cholesterol; LDLC, low-density lipoprotein cholesterol; FPG, fasting plasma glucose; TG, triglycerides; TFA, total abdominal fat area; SFA, subcutaneous fat area; SSFA, superficial subcutaneous fat area; DSFA, deep subcutaneous fat area; VFA, visceral fat area. *There was a significant correlation after a false discovery rate adjustment

**Table S5**. Partial correlation coefficients between abdominal fat areas and metabolic syndrome components in post-menopausal overweight women, excluding participants with high leverage value.

|  |  | SBP | DBP | MAP | TC | HDLC | LDLC | TG | FPG |
| --- | --- | --- | --- | --- | --- | --- | --- | --- | --- |
| TFA | Age | 0.14 (0.02) | 0.01 (0.0001) | 0.11 (0.01) | -0.14 (0.02) | -0.08 (0.006) | -0.15 (0.02) | 0.13 (0.02) | 0.14 (0.02) |
| (n=85) | BMI | 0.09 (0.008) | -0.13 (0.02) | -0.06 (0.004) | 0.02 (0.0004) | -0.23 (0.05) | 0.01 (0.0001) | 0.28 (0.08) | 0.15 (0.02) |
|  | Age and BMI | -0.09 (0.008) | -0.13 (0.02) | -0.07 (0.005) | 0.01 (0.0001) | -0.23 (0.05) | -0.01 (0.0001) | 0.28 (0.08) | 0.14 (0.02) |
| SFA | Age | 0.09 (0.008) | -0.05 (0.003) | 0.08 (0.006) | -0.20 (0.04) | -0.01 (0.0001) | -0.20 (0.04) | -0.04 (0.002) | 0.06 (0.004) |
| (n=85) | BMI | -0.08 (0.006) | -0.16 (0.03) | -0.09 (0.008) | -0.19 (0.04) | -0.11 (0.01) | -0.17 (0.03) | 0.01 (0.0001) | -0.01 (0.0001) |
|  | Age and BMI | -0.08 (0.006) | -0.16 (0.03) | -0.09 (0.008) | -0.19 (0.04) | -0.11 (0.01) | -0.17 (0.03) | 0.01 (0.0001) | -0.01 (0.0001) |
| SSFA | Age | 0.09 (0.008) | 0.01 (0.0001) | 0.12 (0.01) | -0.16 (0.03) | -0.04 (0.002) | -0.08 (0.006) | -0.07 (0.005) | 0.08 (0.006) |
| (n=84) | BMI | -0.03 (0.0009) | -0.08 (0.006) | 0.03 (0.0009) | -0.06 (0.004) | -0.13 (0.02) | 0.02 (0.0004) | 0.04 (0.002) | 0.12 (0.01) |
|  | Age and BMI | -0.02 (0.0004) | -0.08 (0.006) | 0.03 (0.0009) | 0.06 (0.004) | -0.13 (0.02) | 0.02 (0.0004) | 0.04 (0.002) | 0.12 (0.01) |
| DSFA | Age | 0.17 (0.03) | -0.05 (0.003) | 0.06 (0.004) | -0.30 (0.09) * | 0.02 (0.0004) | -0.33 (0.11) * | -0.04 (0.002) | 0.02 (0.0004) |
| (n=84) | BMI | 0.03 (0.0009) | -0.15 (0.02) | -0.06 (0.004) | -0.22 (0.05) | -0.05 (0.003) | -0.25 (0.06) | 0.03 (0.0009) | 0.01 (0.0001) |
|  | Age and BMI | 0.03 (0.0009) | -0.15 (0.02) | -0.06 (0.004) | -0.22 (0.05) | -0.05 (0.003) | -0.26 (0.07) | 0.03 (0.0009) | -0.01 (0.0001) |
| VFA | Age | 0.17 (0.03) | 0.10 (0.01) | 0.16 (0.03) | 0.06 (0.004) | -0.16 (0.03) | 0.04 (0.002) | 0.29 (0.08) * | 0.23 (0.05) |
| (n=83) | BMI | 0.06 (0.004) | 0.07 (0.005) | 0.09 (0.008) | 0.18 (0.03) | -0.23 (0.05) | 0.14 (0.02) | 0.38 (0.14) * | 0.25 (0.06) |
|  | Age and BMI | 0.04 (0.002) | 0.06 (0.004) | 0.08 (0.006) | 0.16 (0.03) | -0.23 (0.05) | 0.13 (0.02) | 0.39 (0.15) * | 0.24 (0.06) |

Value in bracket is presented as R^2^. BMI, body mass index; SBP, systolic blood pressure; DBP, diastolic blood pressure; MAP, mean artery pressure; TC, total cholesterol; HDLC, high-density lipoprotein cholesterol; LDLC, low-density lipoprotein cholesterol; FPG, fasting plasma glucose; TG, triglycerides; TFA, total abdominal fat area; SFA, subcutaneous fat area; SSFA, superficial subcutaneous fat area; DSFA, deep subcutaneous fat area; VFA, visceral fat area. *There was a significant correlation after a false discovery rate adjustment
